# Comparison of strain measurement from multimodality tissue tracking with strain-encoding MRI and harmonic ophase MRI in Pulmonary Hypertension

**DOI:** 10.1186/1532-429X-16-S1-O38

**Published:** 2014-01-16

**Authors:** Yoshiaki Ohyama, Bharath Ambale Venkatesh, Elzbieta H Chamera, Monda Shehata, David Bluemke, Joao A Lima

**Affiliations:** 1 Johns Hopkins University, Baltimore, Maryland, USA; 2 Mercy Catholic Medical Center, Philadelphia, Pennsylvania, USA; 3 National Institute of Health, bethesda, District of Columbia, USA

## Background

Right ventriucular (RV) function is the most important determinant of survival in patients with pulmonary hypertension (PH). Strain-encoding (SENC) MRI has been reported to be useful for the quantitative analysis of RV function. Harmonic phase (HARP) method analyzes myocardial deformation from tagging data and has been used for quantification of left ventricular (LV) function in large multi-center trials. Pixel-based multimodality tissue tracking (MTT) is a new noninvasive method for quantification of cardiac deformation from cine image. The aim of this study is to validate bi-ventricular strain measurement by MTT compared to SENC and HARP MRI in PH patients.

## Methods

In 45 subjects (30 PH patients and 15 normal subjects), RV and LV peak global longitudinal strains (Ell) were measured from long axis 4 chamber view using MTT. LV peak global circumferential strains (Ecc) by MTT were measured from short axis. For validation, RV and LV Ell by MTT were compared to measures by SENC-MRI from short axis, and LV Ecc by MTT were compared to measures by short axis tagged MRI analysis (HARP).

## Results

**Figure 1:**
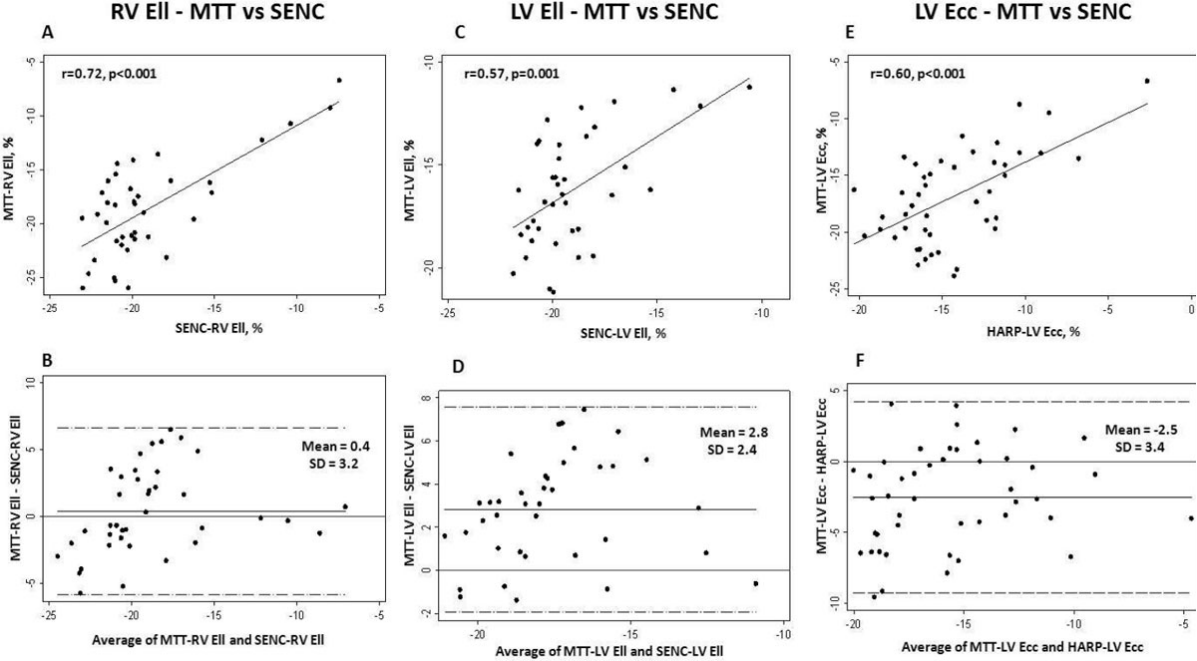
**Correlation and agreement between RV Ell by MTT and SENC-RV Ell (A and B)**. LV Ell by MTT and SENC (C and D), and LV Ecc by MTT and HARP (E and F). The mean difference between the methods and ± SD are indicated.

**Figure 2:**
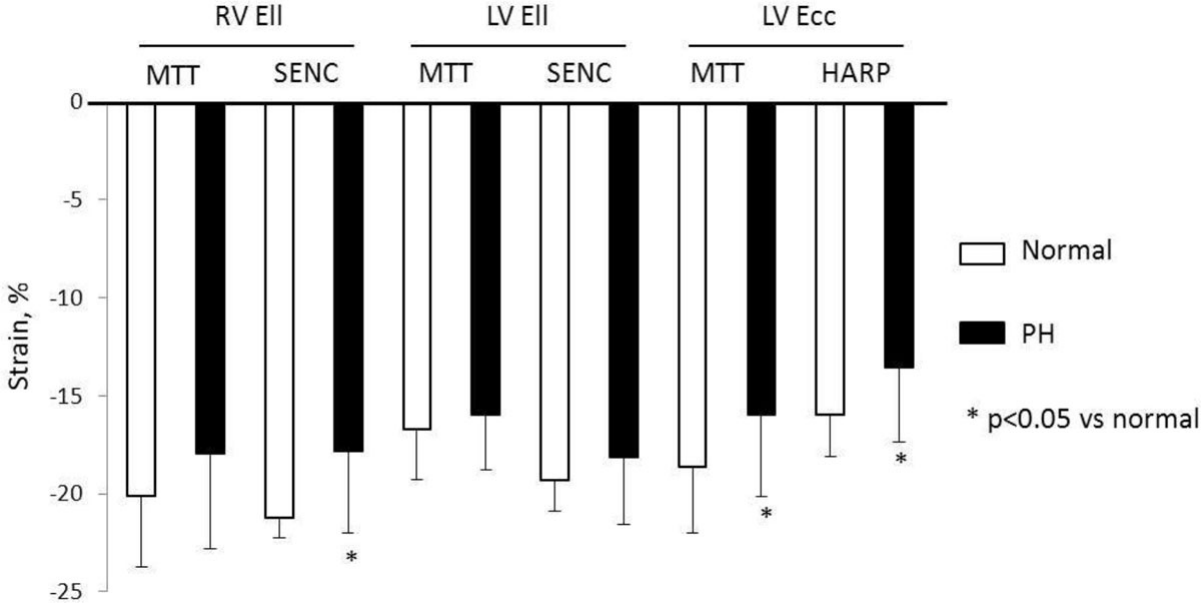
**Comparison of peak strains of PH patients with those of normal subjects in each method**. Values are mean ± SD.

MTT quantified RV Ell correlated closely to those of SENC, with good limits of agreement (-18.7 ± 4.5 vs -19.1 ± 4.8, p = 0.463 for all, r = 0.72, p < 0.001). LV Ell quantified by MTT showed moderate correlation with SENC (-16.2 ± 2.8 vs -19.0 ± 2.4, p < 0.001 for all, r = 0.57, p = 0.001), and LV Ecc by MTT also showed moderate correlation with HARP (-16.9 ± 4.1 vs -14.3 ± 3.5, p < 0.001 for all, r = 0.60, p < 0.001) (Figure 1). PH patients demonstrated reduced RV Ell compared to normal subjects (18.0 ± 4.8 vs -20.1 ± 3.6, p = 0.16 in MTT, -17.8 ± 4.2 vs -21.2 ± 1.0, p = 0.004 in SENC). LV Ecc was also reduced in PH patients compared to normal (-16.0 ± 4.2 vs -18.6 ± 3.4, p = 0.04 in MTT, -13.5 ± 3.8 vs -16.0 ± 2.1, p = 0.03 in HARP) (Figure 2). Strain measurement by MTT showed high reproducibility in both PH patients and healthy subjects.

## Conclusions

We demonstrate that MTT is a reproducible tool for quantification of cardiac deformation using cine images in PH patients. Hence, it could serve as a new rapid and comprehensive technique for clinical assessment of regional cardiac function.

## Funding

Grant sponsor: National Institutes of Health; Grant number: NIH1P50HL084946.

